# Advances in L-Lactic Acid Production from Lignocellulose Using Genetically Modified Microbial Systems

**DOI:** 10.3390/polym17030322

**Published:** 2025-01-24

**Authors:** Lucila Díaz-Orozco, Mario Moscosa Santillán, Rosa Elena Delgado Portales, Luis Manuel Rosales-Colunga, César Leyva-Porras, Zenaida Saavedra-Leos

**Affiliations:** 1 Faculty of Chemical Sciences, Autonomous University of San Luis Potosí (UASLP), San Luis Potosí 78210, Mexico; a261720@alumnos.uaslp.mx (L.D.-O.); mario.moscosa@uaslp.mx (M.M.S.);; 2 Faculty of Engineering, Autonomous University of San Luis Potosí (UASLP), San Luis Potosí 78290, Mexico; luis.rosales@uaslp.mx; 3 Advanced Materials Research Center (CIMAV), Miguel de Cervantes 120, Complejo Industrial Chihuahua, Chihuahua 31136, Mexico; 4 Multidisciplinary Academic Unit, Altiplano Region Campus (COARA), Autonomous University of San Luis Potosí (UASLP), Carretera Cedral km 5+600, Matehuala 78700, Mexico

**Keywords:** lactic acid production, lignocellulosic biomass, agricultural residues, genetically modified microorganisms, sustainable biotechnological processes

## Abstract

Lactic acid is a vital organic acid with a wide range of industrial applications, particularly in the food, pharmaceutical, cosmetic, and biomedical sectors. The conventional production of lactic acid from refined sugars poses high costs and significant environmental impacts, leading to the exploration of alternative raw materials and more sustainable processes. Lignocellulosic biomass, particularly agro-industrial residues such as agave bagasse, represents a promising substrate for lactic acid production. Agave bagasse, a by-product of the tequila and mezcal industries, is rich in fermentable carbohydrates, making it an ideal raw material for biotechnological processes. The use of lactic acid bacteria (LAB), particularly genetically modified microorganisms (GMMs), has been shown to enhance fermentation efficiency and lactic acid yield. This review explores the potential of lignocellulosic biomass as a substrate for microbial fermentation to produce lactic acid and other high-value products. It covers the composition and pretreatment of some agricultural residues, the selection of suitable microorganisms, and the optimization of fermentation conditions. The paper highlights the promising future of agro-industrial residue valorization through biotechnological processes and the sustainable production of lactic acid as an alternative to conventional methods.

## 1. Introduction

Lactic acid is a crucial and versatile organic acid with a wide range of industrial applications in sectors such as food, pharmaceuticals, cosmetics, and biomedicine. It has been reported that D(-)-lactic acid is detrimental to human metabolism, potentially causing acidosis, decalcification, and clinical complications such as diabetes mellitus. Consequently, D(-)-lactic acid is primarily used in chemical industries, while L(+)-lactic acid is preferred in the food and pharmaceutical sectors due to its compatibility with human metabolism. This is because humans possess only the L-lactate dehydrogenase enzyme (*ldhL*), which metabolizes L(+)-lactic acid. In recent years, the demand for lactic acid has grown exponentially, driven by its extensive applications across diverse industries. However, conventional production processes based on refined sugars are associated with high costs and significant environmental impacts. These challenges have prompted the exploration of alternative raw materials and more sustainable production methods. The agri-food industry, one of the world’s most economically significant sectors, is also among the largest generators of organic solid waste. In Mexico, the tequila and mezcal industries produce substantial amounts of agave bagasse, a byproduct of fermentable sugar extraction from agave plants. Agave bagasse is a lignocellulosic residue rich in organic compounds, making it a promising raw material for biotechnological processes. Its high polysaccharide content, derived from lignocellulose, positions it as an ideal candidate for conversion into high-value-added products through microbial fermentation. Lactic acid bacteria (LAB), renowned for their efficiency in lactic acid production, can utilize this agro-industrial waste as a substrate, provided that appropriate pretreatments are applied to release fermentable carbohydrates. This approach highlights the potential of agro-food waste valorization through biotechnological processes, which involve key stages such as residue pretreatment, microorganism selection, process optimization, and product analysis.

In this context, the present review examines the potential of lignocellulosic biomass, particularly agro-industrial residues as substrate for lactic acid production, focusing on three key aspects. First, it discusses the industrial relevance and applications of lactic acid, emphasizing its structure, isomers, and conventional and emerging production methods. Second, it explores the use of lignocellulosic biomass as a substrate, addressing various sources of agricultural residues and by-products, such as sugarcane residues, coconut residues, oil extraction residues, and, in particular, agave residues. Finally, it reviews advancements in the use of genetically modified microorganisms (GMMs) for lactic acid production, with a special focus on genetically engineered *Escherichia coli* strains and their advantages over traditional methods. This analysis aims to provide a complete perspective on the valorization of agro-industrial residues and the development of more sustainable and efficient biotechnological strategies.

## 2. Industrial Relevance and Applications of Lactic Acid

The significance of lactic acid lies in its status as the most widely produced biomolecule, owing to its versatile applications. It serves as a precursor for the production of value-added chemicals across various industrial sectors, including food, cosmetics, pharmaceuticals, textiles, plastics, and chemicals [1,2]. In the food industry, lactic acid plays a pivotal role. It is integral to fermentation processes, the development of probiotics, functional food products, and the production of organic antimicrobial agents used as preservatives [3,4]. In the cosmetics industry, lactic acid has gained prominence as a humectant due to its water-retention capacity, a skin-lightening agent, and a rejuvenator that inhibits tyrosinase activity [5,6]. In the chemical industry, the presence of two reactive functional groups makes lactic acid a highly versatile monomer with significant potential for chemical transformations. It can be converted into valuable industrial products such as propionic acid, acetic acid, and acrylic acid [7]. Table 1 provides additional details on the various applications of lactic acid.

### 2.1. Structure and Isomers

**Figure 1:**
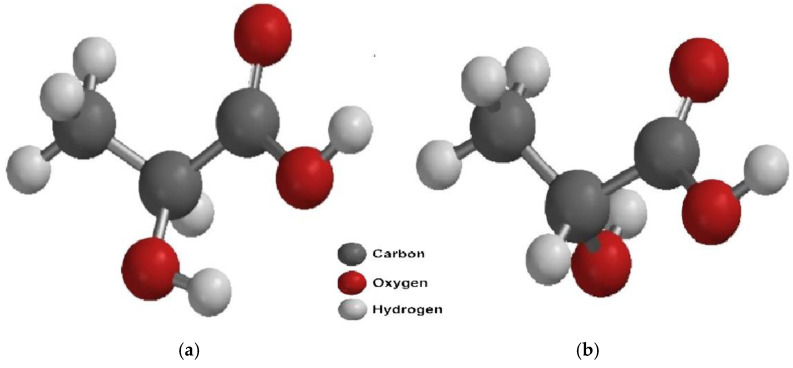
(**a**) Chemical structure of L-lactic acid; (**b**) chemical structure of D-lactic acid.

2-Hydroxypropanoic acid, also known as α-hydroxypropionic acid or commonly referred to as lactic acid, is a natural organic compound. It consists of a hydroxyl group attached to the carbon adjacent to the carboxyl group, making it a carboxylic acid with the formula CH_3_-CHOH-COOH [15,20,21]. Lactic acid is a chiral molecule, meaning it has an asymmetric (chiral) carbon atom, which gives it optical activity. As a result, lactic acid exists as two optically active isomers, known as enantiomers. These enantiomers include the dextrorotatory form, known as L-(+)-lactic acid or S-lactic acid, and the levorotatory form, known as D-(-)-lactic acid or R-lactic acid (Figure 1). Additionally, lactic acid can exist in a racemic form, consisting of an equimolar mixture of the L-(+) and D-(-) isomers, which is optically inactive [3,22,23]. The chemical behavior of lactic acid is determined by its physicochemical properties, such as its acidic nature in aqueous media, the reactivity of its carboxyl and hydroxyl groups, and its optical activity due to the symmetry of the chiral carbon [1]. Lactic acid hydrolyzes into carbon dioxide and water. It is a viscous, hygroscopic liquid with a colorless and odorless appearance. It is soluble in ether, miscible in water and alcohol, but insoluble in chloroform, petroleum ether, and carbon disulfide. Its melting point varies depending on the D and L isomers or the racemic mixture [11,16,23,24]. Table 2 summarizes some physicochemical properties of the lactic acid isomers found in nature.

Lactic acid, as a three-carbon α-hydroxy molecule with two functional groups and a chiral center, exhibits high chemical reactivity. The slightly acidic nature of its carboxyl group and the stereochemistry of its chiral carbon enable lactic acid to undergo various chemical conversion reactions, making it a versatile chemical compound [5,25,26,27]. Lactic acid is an important and multifunctional organic acid with a broad range of industrial applications spanning sectors such as food, pharmaceuticals, cosmetics, and biomedicine [28,29]. Among the isomers, L-(+)-lactic acid is the most widely produced, while D-(-)-lactic acid is less commonly synthesized due to its limited applications [30]. Reports indicate that D-(-)-lactic acid can be harmful to human metabolism, leading to conditions such as acidosis or decalcification. It has even been associated with clinical conditions like diabetes mellitus. For this reason, D-(-)-lactic acid is primarily used in the chemical industry, whereas L-(+)-lactic acid is preferred in the food and pharmaceutical sectors. This preference arises from the fact that humans possess only the L-lactate dehydrogenase enzyme (*ldhL*), which metabolizes L-(+)-lactic acid into pyruvic acid in an NAD^+^ dependent reaction. Additionally, L-(+)-lactic acid is classified as GRAS (Generally Recognized as Safe) by the U.S. Food and Drug Administration (FDA) [12,31,32].

Lactic acid is the simplest hydroxycarboxylic acid and exists as two stereoisomers or enantiomers due to its asymmetric C2 atom. These two forms are optical isomers that rotate light in opposite directions. Typically, an enantiomer that rotates light clockwise is referred to as dextrorotatory (D-(+)), while an enantiomer that rotates light counterclockwise is called levorotatory (L-(-)). Additionally, compounds are classified as D- or L- based on the absolute configuration of D- and L-glyceraldehyde [33,34]. However, lactic acid is an exception to these rules, as it exists as a D-levo (-) isomer and an L-dextro (+) isomer [33]. Both enantiomers share similar physical and chemical properties. There are three types of lactic acid: L-lactic acid, D-lactic acid, and DL-lactic acid (a racemic mixture of the D and L isomers) [30,35]. The racemic mixture is typically produced through chemical synthesis, whereas optically pure L- or D-lactic acid can be obtained via microbial fermentation using renewable resources, depending on the selected strain. Therefore, fermentation is the most suitable method for producing pure L-(+)-lactic acid or D-(-)-lactic acid. The ability to produce specific stereoisomers of lactic acid is widely regarded as a taxonomic characteristic of lactic acid bacteria, allowing for the selection of species or strains that produce only L-(+)- or D-(-)-lactic acid [36]. The two optically active forms, as well as the racemic form, are found in the liquid state, being colorless and water-soluble. In their pure form, they are highly hygroscopic solids with low melting points, whereas the boiling point of the anhydrous product is high. Table 3 provides an overview of the physical properties of each lactic acid isomer and the racemic mixture.

In the health field, it has been reported that both isomers participate in metabolic acidosis [37,38]. In humans, L-lactic acid is an organic acid generated from carbohydrate metabolism under anaerobic conditions, a process known as the Cori cycle or lactic acid cycle. This metabolic pathway connects the muscles and liver. During intense exercise, muscles produce lactate from glucose under anaerobic conditions. The lactate is then transported to the liver, where it is converted back into glucose through gluconeogenesis, and this glucose is returned to the muscles as an energy source. This cycle prevents the accumulation of lactic acid and helps maintain energy levels, although it consumes more energy in the liver than it produces in the muscles [38]. Similarly, DL-lactic acid is a three-carbon, unsaturated, non-volatile carboxylic acid produced in the anaerobic environment of the gastrointestinal tract through bacterial fermentation. Under stable conditions, DL-lactic acid does not enter systemic circulation but is saturated with short-chain volatile fatty acids. However, when fermenting bacteria in the gut have access to an excess of fermentable carbohydrates, DL-lactate can accumulate and enter systemic circulation. While L-lactic acid is readily metabolized to pyruvate in the liver and kidneys, preventing its accumulation in the blood, D-lactic acid is metabolized more slowly and can accumulate to neurotoxic levels [33]. As a result, D-lactic acid has been closely linked to clinical conditions such as diabetes mellitus. Due to these properties, the food and pharmaceutical industries use only L-lactic acid, as the D-lactic acid enantiomer cannot be easily metabolized by humans. However, in the cosmetics industry, a racemic mixture of DL-lactic acid can be used, while in the chemical and textile industries, D-lactic acid finds specific applications [1,6,39].

### 2.2. Lactic Acid Production

The industrial applications of lactic acid span several sectors, including food, pharmaceuticals, medicine, cosmetics, chemicals, and textiles, among others [40,41]. Furthermore, approximately 85% of lactic acid demand is driven by the food industry, while non-food industrial applications account for only 15% of the total demand [15]. Lactic acid can be produced either through chemical synthesis from hydrocarbon-based sources or microbial fermentation using biotechnological routes. The chemical synthesis of lactic acid typically involves the following steps: Hydrogen cyanide is added to acetaldehyde in the presence of a base to produce lactonitrile (this reaction occurs in a liquid phase under high pressure). The lactonitrile is extracted by distillation. It is hydrolyzed in the presence of concentrated HCl and/or H_2_SO_4_ to produce the corresponding ammonium salt and lactic acid. Lactic acid is esterified with methanol, then hydrolyzed with water in the presence of an acid catalyst to produce pure lactic acid while recycling methanol [14,24,31]. However, chemical synthesis is expensive, relies on petrochemical by-products, and produces a racemic mixture of DL-lactic acid, which is undesirable for many applications [2,40,42]. While DL-lactic acid is always produced through chemical synthesis, optically pure L-(+) or D-(-) lactic acid can be obtained through microbial fermentation by carefully selecting the appropriate microorganism [32,43]. Microbial fermentation offers several advantages, such as the production of pure isomers, the use of agro-industrial waste as fermentation substrates, the use of specific microorganisms tailored to each lactic acid isomer, low raw material costs, low energy consumption, and high product yield [44]. Microbial fermentation is the preferred method for obtaining lactic acid from carbohydrate sources, with a contribution of about 90% of the total commercial lactic acid production [5]. Common raw materials include hexose and pentose sugars derived from corn syrups, molasses, beet extracts, whey, and various starches, collectively referred to as agro-industrial waste [45,46]. Currently, glucose and sucrose are the primary feedstocks, although non-edible cellulose holds significant potential as a key substrate in the chemical industry [47]. Microbial fermentation of lactic acid provides the added advantage of recycling agro-industrial waste and by-products as substrates, which not only reduces environmental pollution but also lowers production costs and energy consumption [44,48].

The production of lactic acid through fermentation typically involves four main stages: biomass pretreatment, saccharification to release fermentable sugars, fermentation by specific microorganisms, and the recovery and purification of lactic acid [49]. These stages are briefly described below. Biomass pretreatment can be performed using various techniques [50]: physical methods (milling and extrusion) [51,52], chemical methods (treatment with alkalis, acids, and organic solvents) [52,53], physicochemical methods (autohydrolysis, hydrothermolysis, oxidation, and pyrolysis) [52,54], electrical methods (pulsed electric fields) [55], and biological methods (fungi and bacteria) [56,57]. The choice of pretreatment technique depends on several factors, such as the type of biomass. Physical and chemical characteristics will determine the most effective process. For example, lignocellulosic biomass, such as woody residues, has a rigid structure and requires more intensive methods, such as chemical or thermal treatments, to break lignocellulosic bonds, given the high content of hemicellulose and lignin. In contrast, lignocellulosic biomass like agave residues requires less invasive methods due to its higher composition of cellulose and hemicellulose, which have a less rigid structure [23,58]. Other factors to consider include the pretreatment objective (e.g., for the production of biofuels or value-added products such as organic acids, specifically lactic acid) and the scalability and cost of the method, as the processes must be economically viable.

The second stage, saccharification, commonly involves techniques such as enzymatic, acidic, or alkaline hydrolysis to release fermentable sugars from polysaccharides. Acid hydrolysis typically occurs at pH values below 7, generally between pH 0 and 3, while base hydrolysis typically occurs at pH values above 7, generally between pH 10 and 14, depending on the molecule being hydrolyzed [59,60]. Enzymatic hydrolysis is a widely used operation to break down polysaccharides, such as lignocellulose, into monosaccharides. It offers advantages over other methods, such as acidic hydrolysis, and is, therefore, the most commonly employed technique for lactic acid production [33,61,62].

The third stage is the fermentation of the hydrolysate by selected microorganisms to produce pure lactic acid (L or D isomers). Homofermentative methods are preferred for industrial production, as they result in high yields of optically pure product with minimal by-products. The genera that produce the most lactic acid are *Lactobacillus*, *Streptococcus*, and *Sporolactobacillus* [31]. Likewise, lactic acid bacteria are the most commonly used and include the genera *Lactobacillus*, *Bacillus*, and *Enterococcus* as well as some molds such as the genus *Rhizopus* [49]. Microbial fermentation derived lactic acid typically consists of 99.5% of the L-isomer and only 0.5% of the D-isomer [63]. The fermentation process generally lasts between 12 and 48 h at temperatures ranging from 20 to 35 °C, before the microorganism enters the death phase [64,65]. During this stage, lactic acid is produced as a primary metabolite, alongside microbial growth, until the fermentable sugar is depleted or the pH drops below 3.5 [49].

In the fourth stage, the lactic acid produced is separated from the fermentation medium by removing proteins, sugars, and other unused compounds using techniques such as centrifugation, filtration, and solvent extraction. The lactic acid is then recovered through methods such as purification via precipitation, ion exchange, affinity chromatography, electrodialysis, or membrane filtration [26,45]. Table 4 summarizes pretreatment techniques based on biomass sources.

## 3. Lignocellulosic Biomass as a Substrate for Lactic Acid Production

The sustainable management of agricultural waste is increasingly recognized as a crucial element in global efforts to balance environmental conservation with economic development [70]. Renewable resources such as biomass are key contributors to achieving net-zero carbon emissions [71]. Biomass is the most abundant raw material globally, with an estimated annual production of approximately 181.5 billion tons [72]. As a typical sustainable biological resource, biomass refers to various organisms, including animals, plants, and microorganisms, that enable the conversion of carbon dioxide, water, and sunlight through photosynthesis [41].

**Figure 2:**
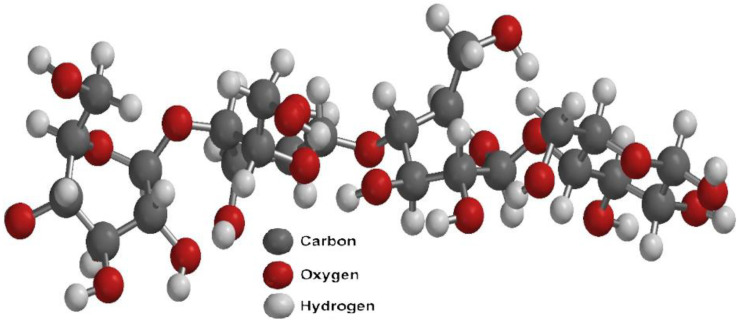
Chemical structure of cellulose I.

Lignocellulosic biomass, also known as natural cellulosic fibers, consists of plant derived materials from cell walls, primarily composed of cellulose (40–50%), hemicellulose (25–30%), and lignin (15–20%), along with minor components like pectin, proteins, salts, and minerals [73,74,75,76]. Cellulose, the main structural component, provides rigidity to plant cells and is the most abundant renewable polysaccharide on Earth [77,78]. Its microfibrils, composed of 500–1400 D-glucose units linked by β-1,4 glycosidic bonds, exhibit a semicrystalline structure with crystalline and amorphous domains [29,79,80,81]. Figure 2 shows the chemical structure of cellulose.

**Figure 3:**
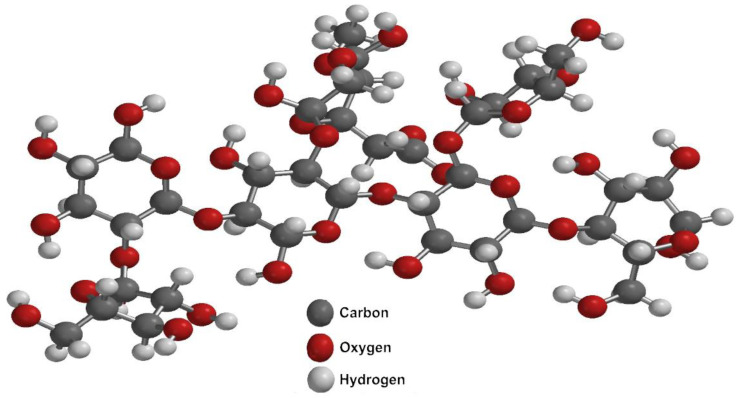
Chemical structure of hemicellulose.

**Figure 4:**
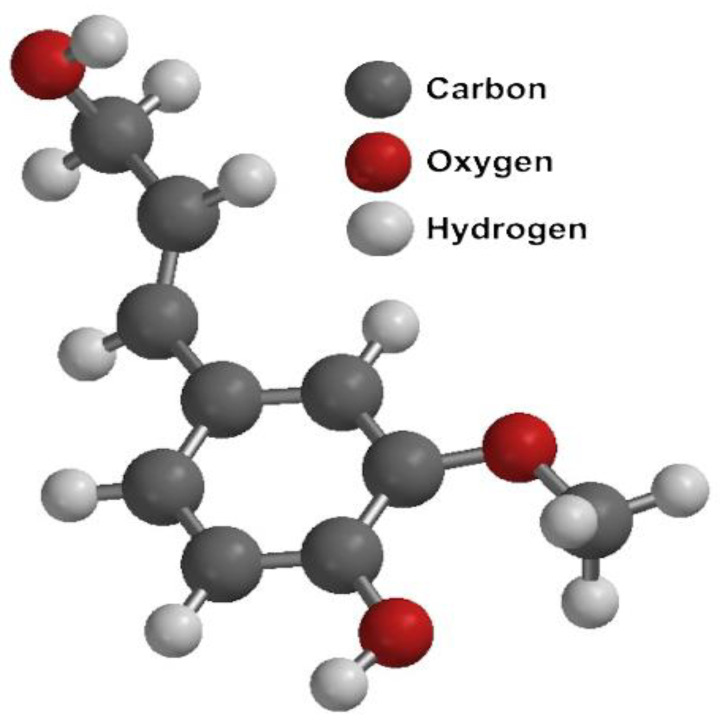
Chemical structure of lignin.

Cellulose exists in polymorphic forms: cellulose I (natural, highly crystalline, and resistant to degradation), cellulose II (less organized and chemically treated for industrial applications), cellulose III (formed via amine treatment), and cellulose IV (produced by heating cellulose III with glycerol) [80,82,83,84,85]. The structure and properties of these polymorphs depend on pretreatment methods, which influence their crystalline or amorphous nature [83,86]. Therefore, the fundamental component of the lignocellulosic complex is cellulose, which forms a skeletal structure throughout the cell wall. The internal spaces are also filled with binding compounds, such as hemicellulose and lignin (Figure 3 and Figure 4).

Lignin, a phenolic polymer, complements cellulose and hemicellulose in the cell wall, forming a nanoscale lignin–carbohydrate network. Its composition varies by plant type: guaiacyl and hydroxyphenyl lignin in gymnosperms, syringyl-rich lignin in angiosperms, and guaiacyl-dominant lignin with some syringyl in grasses [62,87,88,89,90]. This diversity arises from differences in biosynthetic enzymes and polymerization processes [86,91]. Together, cellulose, hemicellulose, and lignin create a complex, regulated structure that provides plants with mechanical strength and resilience.

The most common lignocellulosic biomass includes forest wood residues, agricultural waste, industrial byproducts, and municipal solid waste [57]. Lignocellulosic biomass can be divided into two main categories: woody biomass waste and non-woody biomass waste. Woody biomass includes forest residues such as spruce and cedar trees, wood chips, and sawdust, among others [92]. Woody biomass is the most prevalent organic material on Earth and represents a major renewable energy source. It contains varying compositions of cellulose, hemicellulose, and lignin, depending on the tree species, and accounts for approximately 90% to 95% of all cellulose pulp produced [93,94]. The advantages of using non-woody lignocellulosic biomass as a cellulose source include low cost, availability, abundance, and waste reduction that would otherwise result from landfilling or incineration [95,96]. Non-woody biomass refers to all lignocellulosic biomass sources other than woody plants. Non-woody cellulose sources are classified into three main categories based on their origin: agricultural byproducts, industrial and municipal lignocellulosic waste, and energy crops. Agricultural byproducts refer to the non-edible parts of crops left after harvesting and processing crops such as rice, corn, and wheat [97,98]. These byproducts include primary or field residues generated during harvesting, such as stalks, leaves, and husks, and secondary residues, which are byproducts simultaneously formed during crop processing, such as molasses, bagasse, and pulp [99,100]. Municipal waste, also known as municipal solid waste (MSW), refers to various types of waste generated in urban areas, including household, commercial, and food industry lignocellulosic waste. Municipal waste includes a wide range of materials such as paper waste, cardboard, food scraps, garden waste, and textile residues. Notably, the food industry generates a significant amount of waste, including various byproducts and residues that can potentially be used as a cellulose source [101,102]. In energy crops, bacterial or microbial cellulose differs from plant-derived cellulose due to its higher purity, strength, moldability, and water retention capacity. Most bacteria in their natural habitats generate extracellular polysaccharides such as cellulose, which form protective layers around their cells [103,104]. Bacterial cellulose is a polymer obtained through fermentation using microorganisms of the genera *Acetobacter*, *Rhizobium*, *Agrobacterium*, and *Sarcina*, with *Acetobacter xylinum* being the most efficient species. This polymer shares the same chemical structure as plant-derived cellulose but differs in conformation and physicochemical properties, making it attractive for various applications, especially in the fields of food, separation processes, catalysis, and medicine, thanks to its biocompatibility [105].

**Figure 5:**
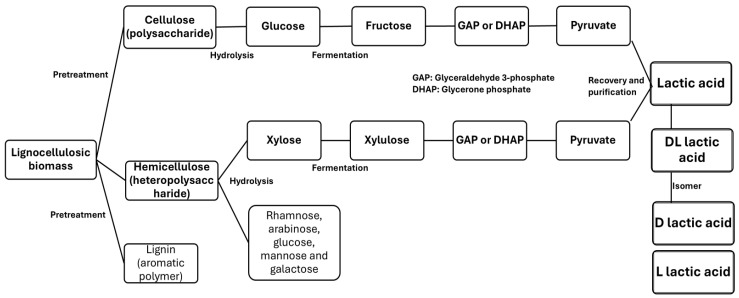
General process for obtaining lactic acid from lignocellulosic biomass.

The organic nature of lignocellulosic biomass makes it valuable through two main biotechnological conversion routes: (i) sugar hydrolysis and fermentation, and (ii) carboxylic acid production via anaerobic fermentation [10,15]. When lignocellulosic biomass is valorized through sugar hydrolysis, most fermentative microorganisms can readily convert glucose into biofuels and biochemical products, such as bioethanol and lactic acid, among others [57,106,107]. In anaerobic fermentation for carboxylic acids, such as lactic acid, research focuses particularly on optimizing traditional anaerobic bioprocesses dedicated to biogas or carboxylate production [108,109]. Therefore, lignocellulosic biomass serves as an excellent source for lactic acid and other products due to its composition, which is rich in fermentable carbohydrates, and its abundance as a renewable resource. The use of specific microorganisms also enhances sustainability and waste reduction, making it key for eco-friendly industrial processes. Figure 5 shows a flow diagram of the general process for obtaining lactic acid from lignocellulosic biomass.

### 3.1. Agricultural Residues and By-Products for Lactic Acid Production

Waste can be classified into those generated by fishing, agricultural, forestry, poultry, and livestock activities, including the disposal of inputs used in these processes, among which agro-industrial residues are included [110]. Specifically, in agricultural industries, waste is classified into two main types: agricultural residues and industrial residues [111]. Agricultural residues are the materials left in the field after crop harvesting (leaves, stems, and seed pods), while industrial residues are organic by-products generated during industrial processing (such as cellulose, hemicellulose, lignin, and nitrogen). Similarly, agricultural by-products are secondary materials or products obtained during primary agricultural processes. These by-products are not the primary target of agricultural activities but hold economic, industrial, or environmental value and can be utilized across various sectors. Examples of agro-industrial residues include sugarcane processing waste, oil extraction residues, fruit waste (*Cocos nucifera* L.), and by-products from distillation industries (e.g., tequila production).

#### 3.1.1. Sugarcane Residues

During the processing stages of sugarcane (*Saccharum officinarum*), both harvest residues, such as sugarcane straw, and by-products like bagasse and molasses are generated [112]. Sugarcane bagasse is obtained after a series of milling steps to extract sugars from the cane. For every ton of sugarcane processed, approximately 0.3 tons of bagasse are produced. Bagasse primarily consists of three lignocellulosic polymers: cellulose, hemicellulose, and lignin. Cellulose is composed of D-glucose units linked by β-1,4-glycosidic bonds; due to its high molecular weight, it is indigestible for humans and constitutes about 40–50% of sugarcane residues. Meanwhile, hemicellulose is an amorphous polysaccharide primarily composed of xylose, along with other sugars such as galactose, mannose, arabinose, and rhamnose, accounting for 25–35% of the by-product. On the other hand, lignin is a phenolic macromolecule resistant to enzymatic degradation and represents 20–30% of bagasse [112,113]. Since sugarcane bagasse is a lignocellulosic material, it requires pretreatments such as hydrolysis to break down the polysaccharides and convert them into value-added products [49,112,114]. The polysaccharides resulting from sugarcane bagasse hydrolysis can be fermented to produce lactic acid, which can subsequently be used to synthesize biopolymers such as polylactic acid (PLA) [115]. PLA is widely employed in packaging materials, medical applications, garbage bags, and mulch films. Similarly, the use of sugarcane bagasse for lactic acid production using microorganisms fermentation, highlighting its potential as a cost-reducing lignocellulosic material that can also serve as an energy source (biofuel) [68]. Moreover, sugarcane bagasse is unequivocally lignocellulosic biomass containing approximately 60% carbohydrates, making it a renewable source of fermentable sugars [46]. These sugars serve as raw materials for the fermentative production of various renewable fuels and chemicals, such as lactic acid. However, other types of residues, such as coconut waste, are also considered lignocellulosic biomass.

#### 3.1.2. Coconut Residues

The extensive use of the coconut fruit (family *Arecaceae*, subfamily *Arecoideae*, genus *Cocos*, species *Cocos nucifera* L.) has resulted in a significant amount of biomass, particularly derived from the coconut shell [69,116]. It is estimated that for every kilogram of harvested coconut, approximately 0.6 kg (dry weight) of coconut shell is produced, which is rich in lignocellulosic biomass (cellulose, hemicellulose, and lignin). In the coconut fruit, the fiber content is approximately 30%, the coconut shell accounts for 40%, and the remainder is ash [117]. Coconut fiber contains cellulose and hemicellulose, which can be utilized for bioethanol production. In terms of chemical composition, coconut fiber predominantly contains cellulose (43.4%), hemicellulose (19.9%), and lignin (45.8%) [61]. Furthermore, the coconut shell contains a substantial number of lignocellulosic polymers, which can be used as a substrate for the hydrolysis of sugars to produce organic acids, biofuels, and bioplastics [49]. One of the most prominent biodegradable plastics is PLA, which is synthesized via the condensation polymerization of lactic acid. The use of coconut shell powder in polylactic acid-based biocomposites have demonstrated that PLA biocomposites with coconut shell degrade faster than pure PLA biocomposites [118].

#### 3.1.3. Residues from Oil Extraction

The production of polylactic acid can also be achieved using other carbohydrate sources, such as residues generated from palm oil extraction. The extraction processes of palm oil (*Arecaceae* family, *Coryphoideae* subfamily, *Elaeis* genus, *Elaeis guineensis* species) generate substantial wastewater and solid residues (biomass) [119]. Palm oil solid residues are primarily lignocellulosic materials, such as cellulose, hemicellulose, and lignin, in varying compositions. These solid residues include empty fruit bunches (EFB), oil palm fronds (OPF), pressed palm fiber (PPF), palm kernel cake (PKC), and oil palm trunks (OPT) [49,120]. The latter contains significant amounts of lignocellulosic polymers (cellulose, hemicellulose, and lignin), and because fermentable sugars can only be obtained from cellulose and hemicellulose, lignin must be removed from the fiber [121]. Palm oil trunk residues have a chemical composition of 43.88% cellulose, 7.24% hemicellulose, 33.24% lignin, 1.01% ash, and 2.73% extracts [122]. Most of the palm oil residues are utilized as commercial solid fuels to generate sustainable and renewable energy due to their high calorific values [120]. Additionally, they serve as potential raw materials for the biochemical, biofuel, and bioplastic industries. The fermentative production of lactic acid from oil palm trunk residues using enzymatic hydrolysis and hydrothermal treatment processes, as these residues contain high glucose content that can be fermented into ethanol or lactic acid—a precursor for synthesizing polylactic acid (PLA), a versatile bioplastic among various biopolymers [123]. Lactic acid has broad applications in multiple industries, especially in the production of PLA for biodegradable plastics [124]. The use of empty fruit bunches of palm oil for lactic acid production, is greatly influenced by operational parameters such as temperature, reaction time, biomass loading, and catalyst concentration on lactic acid yield using barium hydroxide as a catalyst. An improved pretreatment and fermentation method for lactic acid production, employs immobilized cells for efficient recovery of fermentable sugars from palm oil kernel cake [125]. The effect on the fermentation performance of *Actinobacillus succinogenes* in converting fermentable sugars into lactic acid, results in high lactic acid production using immobilized cells with activated carbon and coconut shell.

#### 3.1.4. Agave Residues

The production of lactic acid from fermentable sugars can also be achieved using lignocellulosic residues generated by the tequila industry, such as agave residues [126]. Agave is primarily used for producing both non-distilled alcoholic beverages, like pulque, and distilled spirits, including tequila, mezcal, bacanora, raicilla, and sotol, among others [127]. This production is a longstanding tradition and a significant part of the Mexican economy. However, the industry generates agro-industrial by-products such as bagasse, leaves, and fibers (from leaves and stems), which have been underutilized. According to the Food and Agriculture Organization (FAO), approximately 70 million tons of agri-food residues are generated annually. In Mexico alone, approximately 37.5 million tons of agro-industrial by-products are produced each year [128]. Data from the Tequila Regulatory Council (CRT) indicate that during 2023, 2.288 million tons of agave were required to produce 599 million liters of tequila with a 40% alcohol volume. Consequently, bagasse production reached approximately 671 thousand tons. Bagasse represents 40% of the original weight of the agave stem and is mainly composed of cellulose, hemicellulose, lignin, xylan, and glucan [127]. The percentage of these polysaccharides for *Asparagaceae* family species, such as *Agave tequilana*, *Agave salmiana*, *Agave americana* and *Agave durangensis*, is presented in Table 5.

**Figure 6:**
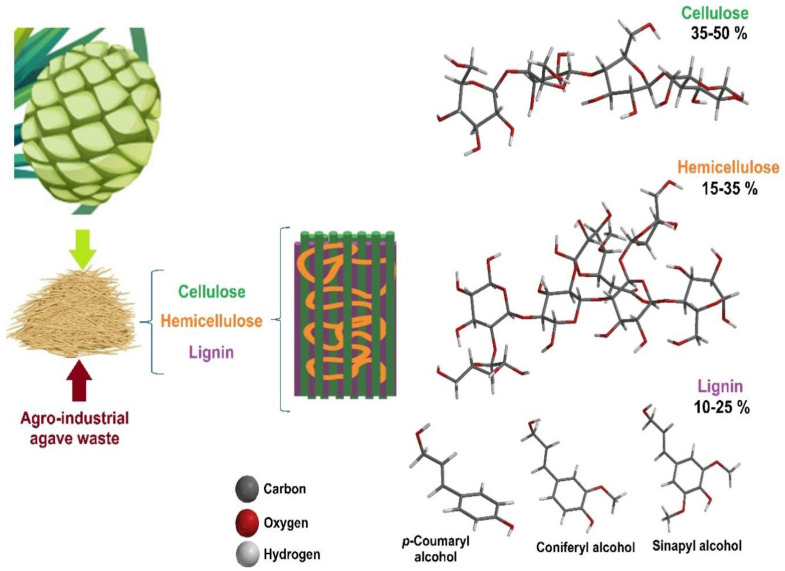
Chemical structure of lignocellulosic biomass of agave.

Cellulose accounts for 40–50% of the agro-industrial by-product [126]. Hemicellulose comprises various carbohydrates, including xylose, arabinose, galactose, mannose, glucose, and uronic acid, connected through glycosidic bonds, representing 25–30% of the by-product [113,126]. Lignin is a complex aromatic polymer and constitutes 15–20% of the residue [126,130]. Xylan is a polysaccharide consisting of a linear chain of xylose residues, while glucan is formed by D-glucose monomers [131]. Figure 6 illustrates the structure of agave biomass.

## 4. Genetically Modified Microorganisms (GMMs) Used in Lactic Acid Production

Lactic acid bacteria (LAB) are microorganisms capable of producing large amounts of lactic acid from fermentable sugars, such as monosaccharides (glucose, fructose, and galactose) and disaccharides (sucrose, maltose, and lactose), requiring nutrients like vitamins, minerals, fatty acids, amino acids, peptides, and nucleotide bases [108]. LAB are microaerophilic or anaerobic and grow at temperatures ranging from 5 to 45 °C, with an optimal pH of 5.5 to 6.5. They are Gram-positive, catalase-negative, and do not form spores. Some lactic acid-producing strains belong to the *genera Leuconostoc*, *Lactococcus*, *Lactobacillus*, *Pediococcus*, *Enterococcus*, *Streptococcus*, *Vagococcus*, *Aerococcus*, *Carnobacterium*, *Tetragenococcus*, *Oenococcus*, and *Weissella*. LAB can be classified into homofermentative bacteria, which produce primarily lactic acid, and heterofermentative bacteria, which produce lactic acid along with other organic compounds and carbon dioxide. Homofermentative bacteria possess the enzyme aldolase and can convert glucose almost exclusively into lactic acid. They typically utilize hexose and pentose sugars (using the glycolysis and pentose phosphate pathways), with a theoretical yield of 1 g/g, producing two molecules of lactic acid per mole of glucose consumed. Some examples of homofermentative lactic acid bacteria include the genera *Streptococcus*, *Lactococcus*, *Enterococcus*, *Pediococcus*, and some *Lactobacillus* strains. On the other hand, heterofermentative bacteria can metabolize glucose into lactic acid, acetic acid, formate, ethanol, and carbon dioxide, among others, using hexose and pentose sugars (through the phosphogluconate and phosphoketolase pathways), with a theoretical yield of 0.5–0.6 g/g. Examples of heterofermentative lactic acid bacteria include the genera *Oenococcus*, *Leuconostoc*, and *some Lactobacillus* strains.

Most LAB present poor fermentative performance and cell viability at low pH, to increase tolerance to low pHs, in addition to specific genetic modifications, other alternatives have been explored, such as: genome shuffling through protoplast fusion, adaptive evolution or combination of both methods [132]. The adaptive laboratory evolution (ALE) applied to LAB strains increased the lactic acid production capabilities, stress tolerance and cell viability [133], by example in recent research, using adaptive evolution improve L-lactic acid fermentation performance from lignocellulose-derived fermentable sugars [134].

Given the complex nutritional requirements of LAB, which complicate industrial processes and increase costs, genetic engineering methods through plasmid transformation could improve the fermentation efficiency in lactic acid production. Some microorganisms, such as *Escherichia coli* and certain yeasts, lack activities for pyruvate formate lyase and lactate dehydrogenase (LDH). These genes can be inserted from L-/D-LDH gene sources from lactic acid bacteria, bovine sources, and fungi, enabling the expression of D(-)-LDH or L(+)-LDH genes to produce D- or L-lactate in minimal media with >99.9% optical purity [4,58,108]. Therefore, in recent years, genetically modified organisms have attracted considerable attention due to their ability to be engineered to meet various production requirements. Additionally, different fermentation methods have varying effects on lactic acid production, especially in fermentation systems designed to maximize both the yield and purity of lactic acid [41,107,135,136].

### Modified Escherichia coli

**Figure 7:**
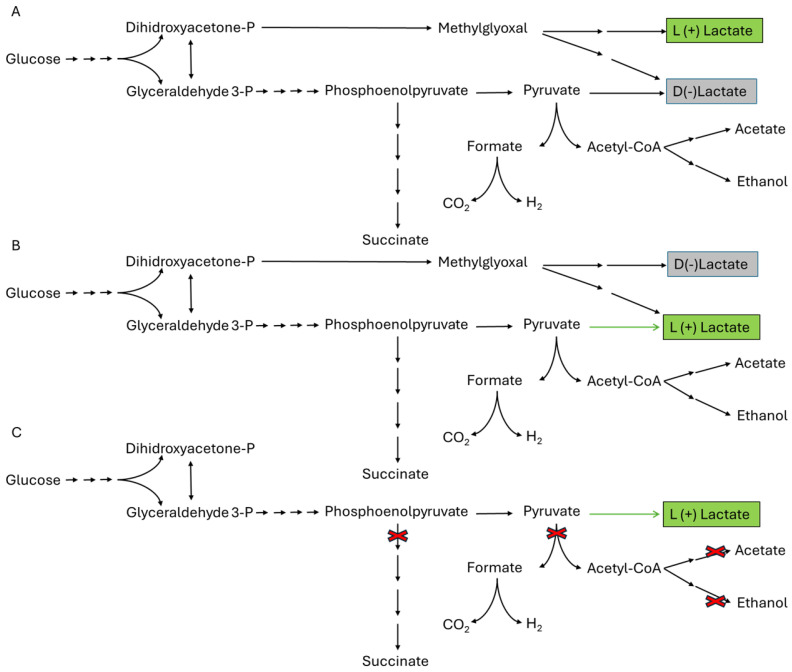
(**A**) Lactate production pathway in wild type *E. coli*. (**B**) Lactate production pathway in *E. coli* mutant strains engineered by replacing native D-LDH with an enzyme to produce L lactate (green arrow). (**C**) Lactate production pathway in *E. coli* mutant strains engineered by replacing native D-LDH with an enzyme to produce L lactate, avoiding the methylglyoxal pathway and redirecting the carbon flux to L Lactate production.

Wild-type *Escherichia coli* is capable of growing and producing lactic acid using hexoses and pentoses via fermentation, resulting in a mixture of organic acids (acetic acid, succinic acid, and formic acid) and ethanol [21,137] (Figure 7A). Additionally, it can grow in media with simpler nutrient requirements compared to conventional lactic acid bacteria. Other advantages of using *E. coli* for lactic acid production are the large number of genetically modified strains that currently exist, which offer great versatility in terms of the use of substrates, in addition to the in-depth knowledge of its genome and genetic regulation; and the wide variety of tools and techniques for its genetic modification, which allows for relatively easy rational genetic modifications, compared to other microorganisms. Genetically modified *E. coli* strains show enhanced lactic acid fermentation efficiency compared to wild-type *E. coli* strains [59,138]. These genetically modified strains have been engineered through (i) replacing the D-LDH gene with L-LDH from lactic acid bacteria, bovine sources, and other sources (Figure 7B). (ii) Preventing the synthesis of racemic mixtures of D- and L-lactate by bypassing the methylglyoxal pathway, thus preventing its accumulation (Figure 7C). (iii) Blocking the aerobic L-LDH to avoid the unwanted utilization of L-lactate or blocking the carbon flux to other fermentation products such as ethanol, succinic, formic, and acetic acids (Figure 7C).

Modified *E. coli* strains can grow and produce L-lactic acid from various disaccharides, including sucrose, and monosaccharides (hexoses and pentoses), such as glucose, xylose, and even glycerol [30,43,109,139,140,141]. Traditionally, lactic acid is produced via the fermentation of carbohydrates by lactic acid bacteria. However, large-scale production using unconventional organisms like genetically modified *E. coli* strains has proven to be a promising alternative due to its high efficiency and versatility [28,137,142,143]. Modified *E. coli* is used to produce lactic acid through the fermentation of sugars like glucose and sucrose. This bacterium has been genetically engineered to eliminate competing pathways and improve fermentation efficiency, allowing for higher lactic acid production. Additionally, modified *E. coli* is more stable and easier to culture than unmodified microorganisms, as it can ferment sugars under anaerobic conditions and produce a single stereoisomer of lactic acid [107,144]. Metabolic engineering has been used to develop *E. coli* strains that produce either D- or L-lactic acid as the fermentation product from different carbon sources, including glucose and xylose, which are present in lignocellulosic hydrolysate syrups [136,145]. This approach achieves high volumetric productivity of lactic acid, with the goal of demonstrating the potential to scale laboratory practices to industrial production. Glucose derived from starch is used for L-lactic acid fermentation by LAB [138,146]. On the other hand, due to its competition with food resources, an alternative non-food substrate, such as cellulosic biomass, is required for L-lactic acid production. However, cellulosic biomass contains significant amounts of xylose, which is not fermentable by most LAB. Therefore, *E. coli* can be employed as an alternative strain for the homofermentative production of optically pure L-lactic acid using cellulosic biomass. Additionally, the production of homolactate through metabolically engineered *E. coli* strains by mutating genes encoding the pyruvate dehydrogenase complex, pyruvate formate lyase, pyruvate oxidase, and phosphoenolpyruvate synthase, redirecting the metabolic flow towards the production of pure lactic acid using glucose as a substrate [147]. Table 6 summarizes some genetically modified *E. coli* strains for lactic acid production.

## 5. Conclusions

In summary, the production of lactic acid from lignocellulosic biomass offers a sustainable and cost-effective solution for the valorization of agro-industrial by-products. By using advanced pretreatment and fermentation technologies, its main components (cellulose, hemicellulose, and lignin) can be exploited to obtain fermentable sugars. This process not only contributes to waste reduction but also provides an eco-friendly alternative to conventional methods of lactic acid production, which rely on refined sugars. The use of genetically modified microorganisms, such as *Escherichia coli*, has proven to be a promising strategy for improving fermentation efficiency and obtaining high-purity lactic acid, potentially leading to more cost-effective and scalable production. Advances in the production of lactic acid from lignocellulose using genetically modified microbial systems have improved the sustainability and efficiency of the process. Strains of microorganisms such as *E. coli* have been developed with optimized metabolic pathways to convert sugars derived from lignocellulose into L-lactic acid with high yield. These strains have greater tolerance to inhibitors generated during pretreatment and the ability to use sugars such as glucose and xylose. In addition, the integration of heterologous enzymes allows a direct conversion of lignocellulose to lactic acid, reducing costs. However, there are still significant challenges regarding the optimization of pretreatments, microbial fermentation improvement, and cost reduction. As biotechnology advances, it is expected that the integration of new developments in genetic engineering, fermentation under more controlled conditions, and the use of alternative substrates like sugar cane, coconut, and oil extraction residues, and agave bagasse will contribute to improving the economic feasibility of these processes. Additionally, the development of more efficient bioprocesses could open new doors for the production of other value-added bioproducts, such as bioplastics and biofuels, using agro-industrial waste. Looking ahead, there is an increased focus on research and optimization of metabolic pathways, as well as improving the stability of microbial strains to scale up lactic acid production sustainably. With the push towards a circular economy and the growing demand for biological and biodegradable products, the valorization of lignocellulosic residues will become a key alternative for a cleaner and more environmentally responsible industry.

## Figures and Tables

**Table 1:** Industrial applications of lactic acid.

Industry	Industrial Application Examples	Type of Isomer (L or D)	Concentrations (Maximums and Minimums)	References
Food	Additive	L-lactic acid	BPF	[1,8,9]
	Flavor enhancer (acidulant)	L-lactic acid	BPF	[8,9,10]
	Preserver	L-lactic acid	BPF	[8,11,12]
	Texturizer	L-lactic acid	BPF	[8,12,13]
	Bacterial inhibitor	L-lactic acid	BPF	[5,8,14]
Cosmetics	Texturizer (rejuvenation)	L-lactic acid or racemic mixture (DL)	0.4–0.9%	[10,12]
	Skin lightener	L-lactic acid or racemic mixture (DL)	0.4–0.9%	[9,15]
	Humectant	L-lactic acid or racemic mixture (DL)	0.4–0.9%	[2,15]
	Anti-acne agent	L-lactic acid or racemic mixture (DL)	0.4–0.9%	[5,15]
Pharmaceutical	Topical ointments	L-lactic acid	BPF 0.4–0.9%	[5,10]
	Parenteral solutions	L-lactic acid	BPF 0.4–0.9%	[1,13]
	Mineral solutions	L-lactic acid	BPF 0.4–0.9%	[1,6]
	Surgical sutures	L-lactic acid	BPF 0.4–0.9%	[5,13]
	Prostheses	L-lactic acid	BPF 0.4–0.9%	[1,16]
Chemical	Production of organic acids: propionic, acetic and acrylic	D-lactic acid or racemic mixture (DL)		[10,17]
	Oxygenated chemicals: esters and propylene glycol	D-lactic acid or racemic mixture (DL)		[1,9,15]
	Polymers: finishing agent in printing materials	D-lactic acid or racemic mixture (DL)		[5,18]
	Cleaning agent	D-lactic acid or racemic mixture (DL)		[5,12]
	Descaling agent	D-lactic acid or racemic mixture (DL)		[12,18]
Textile	Acidulant for deliming hides	D-lactic acid or racemic mixture (DL)		[5,18]
	Cleaning leather hides	D-lactic acid or racemic mixture (DL)		[5,12]
Biopolymers	Material for rigid food and non-food containers	D-lactic acid or racemic mixture (DL)		[19]

**Table 2:** Physicochemical properties of lactic acid isomers.

Property	D(-)-Lactic Acid	L(+)-Lactic Acid	Racemic Mixture (DL)
Melting point (°C)	52.7–54	52.7–54	16.4–18
Boiling point (°C at 1.87 kPa)	103	103	82–122
Viscosity (mPa⋅s)	40.33	40.33	40.33
Density (g/L at 20 °C)	1.249–1.33	1.249–1.33	1.249 1.33
Dissociation constant (pKa at 25 °C)	3.79–3.86	3.79–3.86	3.73–3.79
Heat of fusion (kJ/mol)	11.33	11.33	16.86

Compiled from: [5,24,25,26].

**Table 3:** Physical properties of lactic acid isomers.

Isomer/Property	Melting Point (°C) at 1 atm	Boiling Point (°C) at 1 atm	Solid Density (g/mL) at 20 °C	Liquid Density (g/mL) at 25 °C	Viscosity (mNsm^−2^)	pKa
D(-)	52.8–54	103	1.33	1.057–1.201	40.33	3.79–3.86
L(+)	52.8–54	103	1.33	1.057–1.201	40.33	3.79–3.86
DL	16.8–33	125–140	1.33	1.057–1.201	40.33	3.73

**Table 4:** Processes to produce lactic acid by fermentation.

Biomass Source	Pretreatment Techniques	Saccharification Techniques	Purification Techniques	Microorganism	References
Agave bagasse	Steam explosion	Enzymatic hydrolysis	Filtration and gas chromatography	*Saccharomyces cerevisiae*	[66]
Sugarcane and agave bagasse	Steam treatment	Enzymatic, acid and alkaline hydrolysis	Centrifugation, filtration, and high-performance liquid chromatography (HPLC)	*Saccharomyces cerevisiae*	[67]
Sugarcane bagasse	Steam explosion	Enzymatic hydrolysis	Centrifugation, filtration, and high-performance liquid chromatography (HPLC)	*Bacillus coagulans* DSM2314	[68]
Coconut wastes	Alkaline hydrolysis	Enzymatic hydrolysis	Centrifugation, filtration, and gas chromatography	*Lactobacillus coryniformis* subsp. *torquens* (DSM20004)	[69]
Rice starch	Enzymatic liquefaction	Enzymatic hydrolysis	Centrifugation, filtration, and high-performance liquid chromatography (HPLC)	*Lactobacillus delbuerckii* IFO3202, *Lactobacillus delbrueckii* IFO3534 and *Sporolactobacillus inulinus* ATCC 15538	[30]
Brewer’s grains	Enzymatic hydrolysis	Enzymatic hydrolysis	Centrifugation, filtration, liquid–liquid extraction and high-performance liquid chromatography (HPLC)	*Lactobacillus rhamnosus*	[59]
Sweet sorghum juice	Pressing and extraction	Enzymatic hydrolysis	Filtration, electrodialysis, and anion exchange chromatography	*Bacillus coagulans* A-35	[17]
Woody wastes	Non-isothermal autohydrolysis	Enzymatic hydrolysis	Centrifugation, filtration, and gas chromatography	*Lactobacillus rhamnosus* ATCC7469	[43]
Agro wastes	Acid treatment	Enzymatic hydrolysis	Filtration and spectrophotometry	*Lactiplantibacillus plantarum* and *Lactobacillus brevis*	[11]

**Table 5:** Chemical composition of different species of agave.

Component (%)	*Agave tequilana*	*Agave salmiana*	*Agave americana*	*Agave durangensis*
	Bagasse	Bagasse	Bagasse	Bagasse
Cellulose	41.8–42.0	35.0	40.5	48.0
Hemicellulose	4.4–20	4.6	15-25	20.1
Lignin	7.1–20.1	13.0–19.1	10-15	15.5
Xylan	13.0–19.9	12.0	-	-
Glucan	30.9–45.6	34.1	-	-
Arabinose	0.5–0.9	1.0	-	-

Compiled from: [127,129].

**Table 6:** Genetically modified *Escherichia coli* strains for lactic acid production.

Genetically Modified Strain	Deleted Genes	Introduced Genes	Isomer of Lactic Acid	Lactic Acid (g/L)	Yield (g/g)	Productivity (g/L·h)	Substrate	References
*E. coli* CL3	*ΔpflB, ΔadhE, ΔfrdA, ΔaceF::cat*	-	D-lactate	39.2	0.95	1.31	Glucose	[147]
*E. coli* FBR19	*Δpfl:Cm*, *ΔldhA::Kn*, *ΔfrdABCD*, *Δzce726::Tn10*, *ptsG21*	*ldhL* (plasmid pUCLDH1) from *Streptococcus bovis*	L-lactate	64.3	0.77	-	Glucose and xylose	[148]
*E. coli* FBR11	*ΔpflB*, *ΔadhE*	*ldhL* (plasmid pVALDH1) from *Streptococcus bovis*	L-lactate	63.3	0.78	0.73	Xylose	[149]
*E. coli* JU15	*Δpfl::Cam* *ΔldhA::Kn* *ΔfrdA* *ΔxylFGH E15*	-	D-lactate	34.5	0.84	1.44	Xylose	[150]
*E. coli* LL26	*ΔpflB*, *ΔadhE*, *ΔfrdA*, *ΔldhA* *ΔxylFGH E15*,	*PldhA::IctEBs* from *Bacillus subtilis*	L-lactate	36.96	0.90	1.3	Xylose	[151]
*E. coli* SZ85	*ΔfrdBC*, *ΔackA*, *ΔldhA::ldhL*, *ΔfocA-pflB*, *ΔadhE*	*ldhL* from *Pediococcus acidilactici*	L-lactate	56	0.88	1.2	Glucose and xylose	[152]
*E. coli* TG107	*ΔfrdBC, ΔackA* *ΔldhA::ldhL*, *ΔpflB*, *ΔmgsA*, *ΔadhE*	*ldhL* from *Pediococcus acidilactici*	L-lactate	77	0.85	1.92	Glucose and xylose	[153,154]
*E. coli* WL204	*ΔfrdBC*, *ΔldhA* *ΔackA*, *ΔpflB* *ΔpdhR::pflBp6-acEF-lpd*, *ΔmgsA* *ΔadhE::FRT*	*ldhL* from *Pediococcus acidilactici*	L-lactate	66	0.90	1.09	Xylose	[146]
